# Cognitive Behavior Therapy for Fear of Cancer Recurrence: A Case Study

**DOI:** 10.1007/s10880-018-9545-z

**Published:** 2018-02-21

**Authors:** Marieke van de Wal, Petra Servaes, Rebecca Berry, Belinda Thewes, Judith Prins

**Affiliations:** 1 Department of Medical Psychology, Radboud University Medical Center, P.O. Box 9101, 6500 HB Nijmegen, The Netherlands; 2 Department of Medical Psychology, Maxima Medical Center, Eindhoven/Veldhoven, The Netherlands; 3 Department of Surgery, Canisius-Wilhelmina Hospital, Nijmegen, The Netherlands

**Keywords:** Fear of cancer recurrence, Case study, Cognitive behavior therapy, Oncology, Medical setting

## Abstract

This case study describes the course and content of cognitive behavior therapy (CBT) for clinical fear of cancer recurrence (FCR) in a breast cancer survivor. The CBT for clinical FCR consisted of seven face-to-face therapy sessions and one telephone session. The primary treatment goal was to reduce FCR severity by modifying cognitive processes and dysfunctional behavior. Assessments of FCR and quality of life were completed by the breast cancer survivor pre-therapy, post-therapy, and at 6 and 12 months of post-therapy. In each treatment session, perceived control over FCR was assessed. A clinical nurse specialist participated in evaluation interviews. The patient’s perceived control over FCR increased during the therapy, and FCR severity declined to a non-clinical level. This improvement was still evident at the 6- and 12-month follow-up assessments and was supported by results for secondary and exploratory outcomes measures. FCR offers a great challenge for health care professionals due to the lack of effective treatment options. This case study shows how clinical FCR can be addressed with CBT and can contribute to the improvement of care for cancer survivors.

## Introduction

Fear of cancer recurrence (FCR) is “the fear, worry or concern about cancer returning or progressing” (Lebel, Ozakinci, et al., 2016). Some degree of FCR is considered normal and functional in cancer survivors; it prompts appropriate self-protective responses, such as staying alert for signs of a potential recurrence, and adherence to medical regimens (Lebel, Ozakinci, et al., 2016). However, in 30–70% of individuals, FCR becomes a chronic concern that detrimentally affects their emotional wellbeing, quality of life, and daily functioning (Crist & Grunfeld, 2013; Koch, Jansen, Brenner, & Arndt, 2013; Simard et al., 2013). This fear may persist long after completion of cancer treatment. Severe FCR, also referred to as clinical FCR, does not improve spontaneously with time (Savard & Ivers, 2013; Simard et al., 2013).

Clinical FCR is characterized by the misinterpretation of physical symptoms, the belief that symptoms represent recurrence (Liu et al., 2011), excessive threat-monitoring behavior, frequent seeking of reassurance (e.g., requesting extra medical examinations; Lebel, Tomei, Feldstain, Beattie, & McCallum, 2013), and/or avoidance of situations that remind patients of their disease or treatment (Crist & Grunfeld, 2013; Simard et al., 2013; Thewes, Butow, Bell, et al., 2012). Clinical FCR not only negatively affects the patient but also medical care. It continues to be one of the unmet needs most frequently cited by cancer survivors (Armes et al., 2009). Despite clinical FCR’s high prevalence and unfavorable effect on wellbeing and health care use, adequate psychosocial management of clinical FCR is not routinely available. Furthermore, health professionals acknowledge that FCR is a common problem in clinical practice but are unsure about how to manage it. They often do not refer survivors for extra psychosocial care (Thewes et al., 2014). Evidence-based management strategies for FCR are needed.

We developed an individually delivered cognitive behavior therapy (CBT) program in order to reduce clinical FCR in cancer survivors (van de Wal et al., 2015; van de Wal, Thewes, Gielissen, Speckens, & Prins, 2017). In contrast to two other interventions for the treatment of FCR (Butow et al., 2013, 2017; Lebel et al., 2014), this intervention is developed as an individually delivered blended therapy: a combination of face-to-face contact with e-health or telephone consultations. Whereas the other two therapies rely on cognitive existential therapy (six group sessions; Lebel et al., 2014) or meta-cognitive therapy (five individual sessions; Butow et al., 2013, 2017), the intervention we describe is based on tenets of CBT, and consists of eight therapy sessions. CBT is already an established, effective treatment for anxiety-related disorders in the general population (Hofmann, Asnaani, Vonk, Sawyer, & Fang, 2012); for cancer survivors, individual CBT has proven effective for fatigue (Gielissen, Verhagen, Witjes, & Bleijenberg, 2006), insomnia (Savard, Simard, Ivers, & Morin, 2005), anxiety, and depression (Osborn, Demoncada, & Feuerstein, 2006). One case study showed an effect of CBT on FCR in a cancer survivor with a general anxiety disorder (Montel 2010). We therefore expect CBT to be beneficial for FCR as a standalone problem as well.

In blended therapy part of the therapy is delivered in face-to-face sessions while other parts are given in a different form, in this case e-consultations (with access to a website) or by telephone (and using a workbook). While not much work has been published on blended care, this form of treatment is increasingly being applied by therapists (Wentzel, van der Vaart, Bohlmeijer, & van Gemert-Pijnen, 2016). Face-to-face interventions are relatively costly and resource intensive. There are also barriers that deter patients from engaging in face-to-face therapy, factors such as time demands, reluctance to return to the hospital where cancer treatment took place, having to take time off from work, and travel expenses (Clover, Mitchell, Britton, & Carter, 2015). Blended therapy may overcome some of these barriers because fewer face-to-face sessions are needed, and patients have ongoing access to the website or workbook, which are available to facilitate further skill acquisition and learning. The patient thereby continues treatment between sessions and works on his own mental health, which is beneficial for the development of self-management skills (Wentzel et al., 2016). Due to scarcity of research, it is not yet known whether blended therapy yields benefits comparable to those of face-to-face delivered interventions (van Beugen et al., 2014).

The aim of the current case study was to describe in detail the course and content of blended CBT for clinical FCR in a breast cancer survivor, and to describe ways of overcoming obstacles to successful treatment. The value of case studies in psychology is increasingly recognized; case studies allow in depth description and explanation of intervention feasibility and effectiveness (Yin, 2009). They provide insight into treatment content and symptom changes over time. The present study uses ongoing qualitative and quantitative assessment to support and inform the treatment process.

## Case Presentation

### Medical Background

To assure patient privacy and ensure anonymity, the patient’s personal information has been slightly altered. NG, a 60-year-old Caucasian woman from the Netherlands, was diagnosed with breast cancer (BCa) in the National Breast Cancer Screening Program. Pathological examination revealed a 1.5-cm grade II infiltrating ductal carcinoma, estrogen receptor positive, progesterone receptor, and HER-2/neu negative with an extensive grade II intraductal component. The TNM Classification of Malignant Tumors was pT1cN0(i-)M0, indicating no locoregional spread of the disease or metastasis. In accordance with Dutch oncology guidelines, after breast-conserving surgery to remove the tumor, NG was advised to begin adjuvant radiotherapy, chemotherapy, and endocrine therapy. Four weeks postoperatively, she received 20 fractions of radiotherapy. After being referred to a medical oncologist and much consideration, NG decided to forego chemotherapy because she feared its possible side effects (fatigue and hair loss). She was prescribed Tamoxifen 20 mg daily and was referred for reconstructive surgery. After conserving therapy of the left breast, reduction surgery of her right breast was performed. NG had medical follow-up consultations every 6 months for the first 2 years and an annual mammography.

NG is married, has two children, and three grandchildren. She completed primary education and works as a homemaker. NG has three sisters and two brothers. Her mother, aunt, and two sisters have had breast cancer.

### Clinical Presentation of Psychological Problems

One year after diagnosis, NG felt a new lump in her breast and suspected a recurrence. She contacted the nurse specialist and was invited for an ultrasonography which revealed a cyst. The nurse specialist reassured NG that there were no reasons to suspect a malignancy; in conformance with guidelines, NG was advised to return for her annual clinical examination and mammography in a few months. One month later, NG felt the lump had changed, and so she contacted the nurse specialist again. Pathological examination revealed no signs of malignancy. Shortly after, NG reported a high score of 8 on our measure of stress, the distress thermometer. She also reported concerns regarding body image, FCR, and relationship problems. In the following 2 months, NG telephoned the nurse specialist twice, expressing her worries about a possible recurrence. During these calls, she reported frequent self-examinations and lack of trust in her body. In order to better manage her FCR, NG was then referred to medical psychology.

## Methods

The development of this intervention has been published elsewhere (van de Wal et al., 2015). A call had been sent out to nurse practitioners to refer highly fearful cancer patients for pilot testing of the updated therapy protocol. NG was the first cancer survivor referred to medical psychology. The Cancer Worry Scale was administered to screen for clinical FCR (Douma et al., 2010). Paper-and-pencil assessments took place prior to start of treatment (T0), after completion of treatment (T1), at 6 months (T2), and 12 months (T3) follow-up. All assessments and evaluations were done by an independent researcher in order to prevent potential bias.

### Measures

#### Perceived Control Over FCR: Change Over the Course of Sessions

##### Perceived Control Over FCR Scale

This purpose-designed scale monitors therapy progress, expressed as self-perceived control over FCR. The scale was administered at sessions 1 through 6, and again, after session 8. The instructions asked NG to rate her grip on fear as experienced the past week [“On a scale from 0 to 10, how much perceived control over FCR did you have during the past week?”]. NG recorded her response on a scale ranging from 0 = *no control* to 10 = *maximum control*, with a higher score indicating more perceived control.

#### Primary Outcome: Fear of Cancer Recurrence

##### Cancer Worry Scale (CWS)

This 8-item questionnaire is developed to identify dysfunctional FCR in cancer survivors (Douma et al., 2010). The 8 items of the CWS are rated on a 4-point Likert response scale ranging from 1 = *never* to 4 = *almost always*. Possible scores range from 8 to 32 with a higher score indicating more worries about a recurrence. Typical items are “How often have you thought about your chances of getting cancer (again)?” and “Have these thoughts interfered with your ability to do daily activities?”. NG was asked to restrict her response to how she had felt last week. The CWS has good psychometric properties and is validated in BCa survivors (*α* = 0.87). A cut-off score of ≥ 14 is optimal for detecting high/clinical FCR (Custers et al., 2014).

##### Fear of Cancer Recurrence Inventory (FCRI)

The FCRI provides information on principal characteristics of FCR. Six out of seven subscales were used: severity, triggers, psychological distress, functional impairment, insight, and reassurance seeking. For each subscale, each item is rated on a Likert scale ranging from 0 = *not at all* or *never* to 4 = *a great deal* or *all the time* and NG was asked to limit her response to how she had felt last month.

##### Severity

A 9-item scale that assesses the presence, frequency, intensity, and duration of thoughts associated with FCR. Typical items are “How long have you been thinking about the possibility of a cancer recurrence?” and “Have these thoughts interfered with your ability to do daily activities?” Scores range from 0 to 36 with higher scores indicating more severe FCR. Cronbach’s alpha is 0.88 and 1-month test–retest reliability is 0.87 (Lebel, Simard et al., 2016). A severity score of ≥ 13 is indicative of heightened FCR and a score ≥ 16 can be used to identify survivors who might benefit most from FCR interventions (Simard & Savard, 2009, 2015).

##### Triggers

This 8-item subscale assesses specific situations that make one think about the possibility of cancer recurrence (7-items) and to what degree these situations are generally avoided (1-item). Typical items are “The following situation makes me think about the possibility of cancer recurrence: conversations about cancer or illness in general” and “Generally, I avoid situations or things that make me think about the possibility of cancer recurrence.” Scores range from 0 to 32 with higher scores indicating more sensitivity and exposure to triggers of FCR. Cronbach’s alpha is 0.93 and 1-month test–retest reliability is 0.78 (Lebel, Simard et al., 2016).

##### Psychological Distress

A 4-item subscale that includes items for emotions frequently triggered by thoughts about cancer recurrence. Typical items are “When I think about the possibility of cancer recurrence, I feel: frustration, anger or outrage” and “When I think about the possibility of cancer recurrence, I feel: sadness, discouragement or disappointment”. Scores range from 0 to 16 with higher scores representing more dysfunctional emotions in reaction to FCR. Cronbach’s alpha is 0.88 and 1-month test–retest reliability is 0.79 (Lebel, Simard et al., 2016).

##### Functional Impairment

Includes six items representing domains that can be disturbed by FCR. Typical items are “My thoughts or fears about the possibility of cancer recurrence disrupt: my quality of life in general” and “My thoughts or fears about the possibility of cancer recurrence disrupt: my ability to make future plans or set life goals.” Scores range from 0 to 24 with higher scores indicating more functional impairment due to FCR. Cronbach’s alpha is 0.94 and 1-month test–retest reliability is 0.71 (Lebel, Simard et al., 2016).

##### Insight

Includes three items and assesses the extent to which patients perceive their fear as excessive or unreasonable. Typical items are “I feel that I worry excessively about the possibility of a cancer recurrence” and “I think that I worry more about the possibility of a cancer recurrence than other people who have diagnoses of cancer.” Scores range from 0 to 12 with higher scores indicating more insight into FCR. Cronbach’s alpha is 0.85 and 1-month test–retest reliability is 0.85 (Lebel, Simard et al., 2016).

##### Reassurance Seeking

Includes three items representing reassurance behaviors specific to FCR. Typical items are “When I think about the possibility of cancer recurrence, I use the following strategy to reassure myself: I go to the hospital or clinic for an examination” and “When I think about the possibility of cancer recurrence, I use the following strategy to reassure myself: I call my doctor or another health professional.” Scores range from 0 to 12 with higher scores indicating more reassurance seeking behavior. Cronbach’s alpha is 0.71 and 1-month test–retest reliability is 0.56 (Lebel, Simard et al., 2016).

#### Secondary Outcome: Quality of Life

##### EORTC-QLQ-C30

The European Organization for Research and Treatment of Cancer (EORTC) Quality of Life Questionnaire Core 30 (QLQ-C30) and the BCa module (QLQ-BR23) were completed. The QLQ-C30 consists of one global health status/quality of life scale (GH/QoL), five functional scales, and nine symptom scales. For all scales, NG was asked to reflect over the last week and choose the best answer for how she had felt (symptom and functional subscales) or how she would rate her general wellbeing and quality of life (GH/QoL subscale). Response scales for the symptom and functional scales ranged from 1 = *not at all* to 4 = *very much*, whereas the GH/QoL scale ranged from 1 = *very poor* to 7 = *excellent*. A high score on a functional scale represents a high level of functioning while it indicates greater impairment on a symptom scale (Aaronson et al., 1993; Sprangers et al., 1996).

The GH/QoL subscale consisted of two items; a typical item is “How would you rate your overall health during the past week?” Among the functional scales, the Physical Functioning subscale consisted of five items; a typical item is “Do you have any trouble doing strenuous activities, like carrying a heavy shopping bag or a suitcase?” The Emotional Functioning subscale consisted of four items; a typical item is “Did you feel depressed?” Among the symptom subscales, the Fatigue subscale consisted of 3 items; a typical item is “Did you need to rest?” The Insomnia subscale consisted of one item which was “Have you had trouble sleeping?” Cronbach’s alpha ranged from 0.54 for the role functioning scale to 0.96 for the global health quality of life (GH/QoL) scale (Osoba et al., 1993).

The European Organization for Research and Treatment of Cancer (EORTC) BCa module (QLQ-BR23) was completed by NG as well. The QLQ-BR23 module consists of 23-items covering symptoms and side effects related to different treatment modalities (symptom scales), body image, sexuality, and future perspective (functioning scales). For all scales, NG was asked to indicate the extent to which she had experienced certain symptoms (symptom scales) or problems (functioning scales) during the past week. Response scales ranged from 1 = *not at all* to 4 = *very much* (Sprangers et al., 1996). The Body Image subscale consisted of four items; a typical item is “Did you find it difficult to look at yourself naked?” The Future Perspective subscale consisted of one item which was “Were you worried about your health in the future?” The Breast Symptoms subscale consisted of four items; a typical item is “Was the area of your affected breast oversensitive?” In a Dutch sample of breast cancer survivors the internal consistency was found to be moderate to good (ranging from 0.57 to 0.89) (Sprangers et al., 1996).

#### Exploratory Outcomes

##### Hospital Anxiety and Depression Scale (HADS)

The HADS is a 14-item self-report scale that assesses symptoms of anxiety and depression (Zigmond & Snaith, 1983). A higher score indicates more distress, and a score > 15 indicates a clinical level of distress (Vodermaier & Millman, 2011). NG was asked to select the response option that best described her feelings past week. Response scales ranged from 0 to 3 and verbal anchors differed per item. A typical Anxiety subscale item is: “Worrying thoughts go through my mind” and a typical Depression subscale item is: “I feel as if I am slowed down.”

##### Distress Thermometer (DT)

The DT measures distress severity on a scale from 0 = *no distress* to 10 = *extreme distress* (NCCN, 2003). Instructions for completing the DT are as follows: “Please circle the number (0–10) that best describes how much distress you have been experiencing in the past week including today.” In the Netherlands, the DT is used for routine screening of distress in medical practice. A cut-off score of 5 is ideal to detect clinical distress (Tuinman, Gazendam-Donofrio, & Hoekstra-Weebers, 2008).

##### Satisfaction With Life Scale (SWLS)

The SWLS is a 5-item instrument, with *α* = .87, that provides a global judgment of satisfaction with one’s life; a typical item is “I am satisfied with my life,” and responses are on a scale ranging from 1 = *strongly disagree* to 7 = *strongly agree* and higher scores indicating greater satisfaction (Diener, Emmons, Larsen, & Griffin, 1985).

##### Body Vigilance Scale (BVS)

This 4-item questionnaire includes three items that respectively assess: attentional focus; perceived sensitivity to bodily changes; average duration of time spent attending to sensations; and a fourth item that rates attention paid to 15 bodily sensations, e.g., palpitations. Responses are on a scale ranging from 0 = *not at all*/*never* to 10 = *extremely*/*constantly*, with higher scores indicating greater vigilance. Cronbach’s *α* is 0.82 (Schmidt, Lerew, & Trakowski, 1997; van Laarhoven, Kraaimaat, Wilder-Smith, & Evers, 2010).

##### The Impact of Events Scale (IES)

The 15-item IES was used to assess cancer-specific distress. Instructions to respondents state: “Below is a list of difficulties people sometimes have after a stressful life event such as cancer. Please read each item, and then indicate how distressing each difficulty has been for you during the past 7 days.” The IES provides a total score and scores on two subscales that assess: (1) Intrusion, i.e., the extent to which a cancer survivor experiences intrusive thoughts about cancer for which a typical item is “I thought about it when I didn’t mean to”; and (2) Avoidance, i.e., the tendency to avoid thinking about cancer for which a typical items is “I stayed away from reminders of it.” Responses are on a 4-point scale: 0 = *not at all*, 1 = *rarely*, 3 = *sometimes* and 5 = *often*, with higher scores indicating greater impact, and Cronbach’s alphas of 0.87 for intrusion and 0.82 for avoidance (Corcoran & Fischer, 1994; Horowitz, Wilner, & Alvarez, 1979).

##### Checklist Individual Strength (VVV)

This 4-item short-form measures the experience of fatigue on a 7-point scale ranging from 1 = *I agree* to 8 = *I disagree*, with higher scores indicating greater Fatigue. Instructions state: “Please indicate how you have felt last week”; a typical item is “I tire easily.” Cronbach’s alpha for the VVV is 0.88 (Alberts, Smets, Vercoulen, Garssen, & Bleijenberg, 1997).

##### Life Orientation Test (LOT)

This 12-item questionnaire measures optimistic and pessimistic personality traits. Instructions state: “To what extent do the following items generally apply to you?” and a typical item is “In uncertain times, I usually expect the best.” Responses are recorded on a scale ranging from 4 = *strongly agree* to 0 = *strongly disagree*, and higher score indicates a more positive attitude; Cronbach’s alpha for the LOT has been shown to be 0.76 (Scheier & Carver, 1985).

### Data Analysis

Post-treatment, 6 and 12 months’ FCR follow-up scores were compared to baseline scores and scores of a normative BCa sample. Scores on the CWS were compared to a normative sample of 194 Dutch BCa survivors, mean age 57.0 (SD 10.2) with a mean time since surgery of 4.7 years (SD 2.3) (Custers et al., 2014). FCRI scores were compared to a normative sample of 227 Canadian BCa survivors, mean age 59.0 years (SD 0.6) with a mean time since diagnosis of 4.9 years (SD 0.2) (Simard & Savard, 2009). Reliable change was established using the procedure by Jacobson and Truax (1984) and normative BCa data for the CWS and FCRI (Custers et al., 2014; Simard & Savard, 2009). The reliable change index (RCI) was calculated for the primary outcome, FCR, to determine if reliable change had occurred between baseline and follow-up assessments (Jacobsen & Truax, 1984). The individual RCI for NG was calculated as the difference in raw scores on the CWS or FCRI at baseline and T1, T2, and T3, divided by the standard error of the differences between the scores (van de Wal et al., 2017). A score shows reliable change (improvement or deterioration) when the RCI exceeds the value of 1.96 (Jacobsen & Truax, 1984).

## Course and Content of the Intervention

### Theoretical Model

**Fig. 1:**
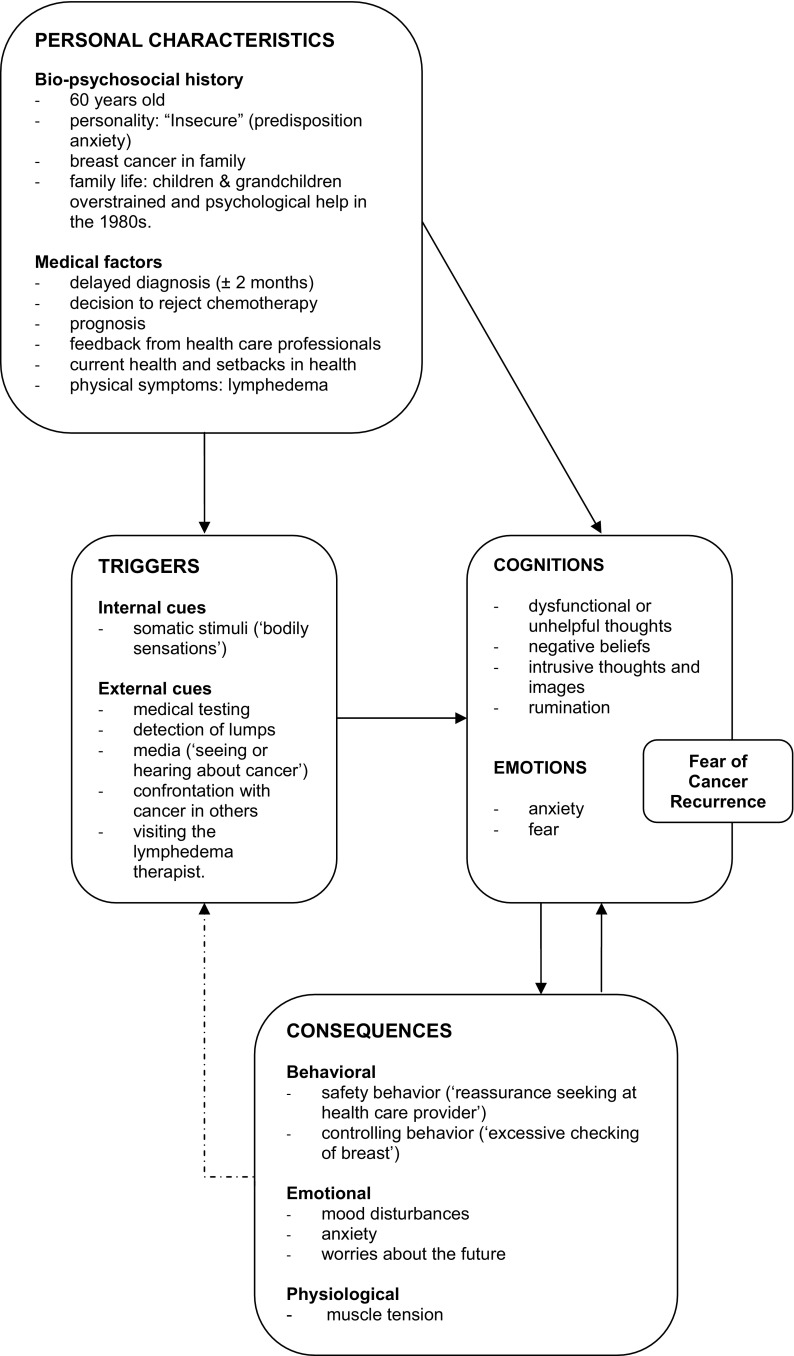
Personalized model of FCR

The intervention follows the theoretical formulation of FCR by Lee-Jones, Humphris, Dixon, & Hatcher (1997). In this model, as shown in Fig. 1, FCR is a distressing *emotion* maintained by dysfunctional *cognitive patterns*, such as recurring unhelpful thoughts, negative beliefs, intrusive images, or persistent rumination. These cognitions cause a person to interpret *events or internal stimuli* as potentially threatening to their health and wellbeing, thereby *triggering* FCR. *Behavioral strategies* that may provide short-term alleviation of fear, such as avoidance, or safety-seeking behaviors, may actually sustain FCR in the long run by preventing changes in cognitive appraisal and/or by providing further exposure to triggers of FCR. CBT targets FCR by changing *dysfunctional cognitive patterns* and *behavioral responses* as specified in this model (Lee-Jones et al., 1997; van de Wal et al., 2015).

This conceptualization of FCR shares similarities with the Health Anxiety Model by Salkovskis and Warwick (2001). For instance, both models share the elements of fear producing stimuli and behavioral consequences of anxiety/fear. However, the FCR model is cancer specific. Criteria of anxiety disorders do not easily apply to clinical FCR in cancer survivors; only the minority of high fearful cancer survivors were found to fulfill the diagnostic criteria for hypochondria, illness anxiety disorder, or generalized anxiety disorder (Dinkel, Kremsreiter, Marten-Mittag, & Lahman, 2014; Simard & Savard, 2015; Thewes et al., 2013). What defines FCR is that highly fearful survivors report intrusive thoughts and symptoms that are mainly related to the specific cancer experience. Survivors do not necessarily worry about their general health but tend to worry specifically about the possibility of a recurrence (Simard & Savard, 2015; Thewes et al., 2013).

### Treatment Manual

This intervention we used combines of face-to-face contact with e-health or telephone consultations. By default, patients are offered the online version of the therapy. In case of computer illiteracy, i.e., no access to a computer, or lack of a skill set to adequately use the computer (Norman & Skinner, 2006), the paper-and-pencil workbook (with DVD) is offered as an alternative.

#### Blended Therapy for FCR

Therapy is delivered by trained psychologists and consists of five individual face-to-face sessions (sessions 1, 2, 3, 5, and 8), with three electronic or telephonic consultations (sessions 4, 6, and 7) within 3 months and one follow-up session 3 months later (session 9). The first four sessions are scheduled weekly, other sessions fortnightly. Between sessions, home assignments are completed using a workbook (with DVD) or the website.

The face-to-face sessions are structured and are carried out according to protocol. With the exception of the first session—which takes 90 min—all other face-to-face sessions last around 45 min. In the first face-to-face session, the therapist gathers a full social and medical history of the patient, identifies the presenting problem (FCR), and sets the stage for the next therapy sessions. E-health/telephone consultations take approximately 15–20 min and are structured in a similar way as the face-to-face sessions. During these sessions, the emphasis is on providing motivational support and giving personalized feedback to ensure that the home assignments are understood.

The primary treatment goal is to reduce the severity of FCR by increasing the sense of control over fear, by modifying cognitive processes and dysfunctional behavior related to FCR. In this case, the patient (NG) was treated by a female therapist. An overview of the treatment content is given in Table 1.

**Table 1:** Content of the intervention by therapy session

Session	Delivery	Week	Time (minutes)	Session components
1	Face-to-face	1	90	Case formulation: a patient’s story Discuss therapy rationale Review FCR and complete a personal FCR model to identify targets for intervention Establish personalized therapy goals Introduce at-home assignments
2	Face-to-face	2	60	Explain the basic tenets of CBT Discuss and visualize the association between thoughts, feelings, and actions Review the concept of helpful beliefs Practice in filling out thought records
3	Face-to-face	3	60	Review the completed thought record(s) to identify unhelpful thoughts and behavioral consequences of FCR Differentiate realistic from unrealistic worries and establish more helpful thoughts Explore and identify dysfunctional behavioral patterns Create a ranked list of situations that induce FCR and propose a behavioral experiment Introduction of a mindfulness or progressive muscle relaxation exercise
4	Telephone	4	15	Review of progress made and problems encountered Encourage at-home practice of relaxation exercises Encourage at-home practice of CBT Psychoeducation on FCR
5	Face-to-face	6	60	Review therapy goals, discuss areas of concern, and make future plans (beyond therapy) Discuss completed thought records and/or behavioral experiments Therapist agrees to/supports patient’s request to have patient’s husband join them for next session in place of the scheduled one-on-one telephone contact (*deviation from protocol*) Therapist supports patient’s decision to forfeit relaxation exercises (*deviation from protocol*)
6	Face-to-face with husband present. Original plan was for telephone contact	7	60	Encourage husband in treatment process Encourage better communication between partners Enlist support from marital partner Strengthen marital relationship Engage patient and partner to make future plans
7	Face-to-face with husband present. Original plan was for telephone contact	9	60	Assess impact of engaging husband in treatment process Encourage further improvement in communication with partner Encourage further support from marital partner Identify and modify unhelpful thoughts Therapist agrees to return to individual sessions
8	Face-to-face	11	60	Review therapy goals, progress made so far and discuss possible future pitfalls Define and finalize the relapse prevention plan Evaluate the therapy process Schedule an appointment for the booster session
9	Face-to-face (booster session)	24	60	Review the FCR model and progress made during therapy Discuss difficult situations and how to overcome them Relapse prevention plan

This table is modeled on the table presented by van de Wal et al. (2015) that describes the study protocol (plan) that guided the intervention

### Content

#### Session 1: Face-to-Face

NG told the therapist that BCa was first tentatively diagnosed 18 months ago; it took 2 months before she received a definite diagnosis. This diagnostic delay caused her a lot of uncertainty and anxiety. Although adjuvant chemotherapy was indicated and advised, NG decided not to have chemotherapy because she was worried about side effects, such as fatigue and hair loss. Even so, she now occasionally worried whether she had made the right choice: “Would chemotherapy have lowered my risk of recurrence?” NG reported thinking daily about cancer recurrence and its consequences. She considered certain events as particularly stressful: the weeks before a medical examination, breast changes, aches or pains, and the diagnosis of cancer in relatives. These events would most certainly trigger FCR. For instance, NG examined her breasts several times a day and any change in the look or feel of her breasts frightened her. For reassurance, she had asked her nurse specialist for extra medical examinations on two occasions in the past 4 months. On both occasions, ultrasound revealed a lymph-filled mass but there was no reason to suspect a recurrence. However, NG was only briefly reassured and further efforts to dismiss FCR were unsuccessful. She felt unequipped to manage FCR herself, but did find some relief in visiting a lymphedema therapist twice a week for manual lymph drainage and remedial exercise therapy, to reduce breast swelling and lymphatic obstruction.

NG experienced mood disturbances, functional impairments and continuously worried about her future. As such, she would not plan any activities ahead. NG described herself as being insecure; she had received psychological therapy in the past. In the 1980s, NG suffered from a maladaptive response to psychological stress whereby she experienced symptoms of mental overload and overstrain (“surmenage”). For this, she had completed a 3-month rehabilitation program (rational emotive therapy). She also expressed concern that, given her family history, there could be a BRCA gene mutation in her family.

At the end of the first session, the therapist helped NG visualize and complete her own personal FCR model on a whiteboard, i.e., a less detailed version of Fig. 1. This important step created a mental framework for NG, a conceptual model of what she was experiencing, a framework to help NG better understand her FCR. More importantly, the personal FCR model helped her to better understand and collaborate with the treatment process, to better define her role and responsibilities as a patient working in collaboration with her therapist. The model served as a guide for subsequent therapy sessions by identifying the most appropriate cognitive and behavioral targets for intervention. NG received education about FCR, e.g., that it’s normal and functional to experience some FCR, but that FCR may become dysfunctional at some point. Additionally, the aim and rationale of CBT were explained (the aim is not to take away all FCR, but rather to reduce its severity to lower, more functional levels). Beside the main objective of reducing FCR severity, NG had formulated three realistic, but rather vague, additional treatment goals for herself: fewer “down” days, fewer worries about the future, and greater optimism. The therapist and patient worked to operationalize these goals by answering the following questions: What would the patient like to happen? What does the patient mean by “down” days? Worries about the future were directly linked to, and therefore combined with, the main therapy goal of reducing FCR. Greater optimism was operationalized as becoming more skilled in formulating helpful, positive, thoughts in response to dysfunctional beliefs. Fewer “down” days would be achieved when there were less daily symptoms of a low mood, e.g., increased activity, less withdrawing from social events and less irritability.

#### Session 2: Face-to-Face

Last week NG had studied automatic, unhelpful thoughts and had learned how to replace them with more helpful ones. In this session, the therapist proposed a three stage ABC model of FCR with activating events (A), beliefs (B), and consequences (C) to explain how the perception of events influences how one feels and acts (Ellis, 1991). It was explained how activating events (e.g., discovering a lump during breast self-exams), in a BCa survivor’s personal model of FCR, can automatically trigger certain beliefs (“This lump feels similar as when I discovered breast cancer…”) and thereby influences how one feels (panic) and acts (call the nurse to request an extra medical check-up). The therapist told NG that we cannot always change the situation that we are in, but that we are able to identify and modify the beliefs we hold and we can come up with alternative, more functional thoughts. In therapy, this is done by completing thought records that incorporate the ABC model. Together the with the therapist, NG completed a thought record describing a recent situation that had caused her fear. NG told she had met a pale, bald boy in a wheelchair after returning home from her first therapy session (A). This encounter left NG sad for the rest of the day and caused her to worry about cancer (C). By dissecting this situation together with her therapist, NG came to recognize that she had unhelpful catastrophizing thoughts (“Oh, this boy has cancer... How terrible!” and “This is not going to end well!…Cancer means death”) that had caused her to feel this way and left her worrying about a possible recurrence. NG agreed to practice completing two thought records (containing steps A, B, and C) in the next week.

#### Session 3: Face-to-Face

After successfully identifying automatic unhelpful cancer-related thoughts during the previous weeks, the therapist introduced the next step in CBT: challenging unhelpful thoughts and replace them with more helpful ones. Because unhelpful recurrence-related thoughts might technically be free of distortions, the focus of this step lies more on the modification of beliefs (coming up with alternative, more helpful thoughts) than trying to dispute the dysfunctional ones. At first, NG was inclined to interpret ambiguous cancer-related information negatively, and she felt over involved in cancer stories from others (“if that woman has a disease recurrence, it will probably happen to me as well”). During this session, NG learned to reframe FCR provoking beliefs into less disturbing ones (“Every medical situation is different. It is not rational to compare hers to mine”). Her personal FCR model helped make clear that her impulsive controlling behaviors were an attempt to manage FCR. This recognition created an opportunity for the therapist to introduce a response prevention exercise. NG created a hierarchy of situations that induced feelings of FCR. Her highest rated situation was her daily breast examination—checking for lumps, swollen nodes, or disfigurement. NG believed that daily breast-exams would help to detect a recurrence early. The therapist suggested daily exposure and response prevention exercises: NG would refrain from breast self-exams in the next 2 weeks, thereby confronting her worst fear. Over time, anxiety would become more manageable and the frequency of breast examinations would decrease.

Mindfulness and relaxation exercises were introduced. Because NG was mainly experiencing physical stress, a quick relaxation technique was preferred over mindfulness. The therapist explained what stress is and how relaxation can counteract the stress response to FCR. In session, NG practiced a progressive muscle relaxation technique that she could easily apply at home when she experienced physical symptoms of anxiety such as muscle tension.

#### Session 4: Telephone

As NG was not sufficiently computer literate for an e-health consultation, a telephone consultation was her preferred option. In this session we evaluated how helpful the exercises were, and if there were any difficulties with at-home practice. NG had practiced the 10-min relaxation exercise but experienced emotional discomfort doing this. The therapist explained to NG that it takes regular practice (also in peaceful circumstances) to achieve greater benefit from relaxation exercises. She was encouraged to try another relaxation exercise at home in the next week.

NG did continue practicing cognitive restructuring and response prevention techniques in everyday situations. Furthermore, instead of actively seeking out cancer-related stimuli she now tried to pay less attention to them. Prior to therapy, she would read the obituary notices in the newspaper daily, searching for information indicating cancer as the cause of death. At this point in therapy, NG realized that her search-behavior merely triggered FCR, upsetting her. NG experienced difficulties in carrying out her exposure and response prevention exercises. While she still felt a strong need to perform breast-exams in front of the bathroom mirror twice a day, NG successfully stopped herself from examining her breasts twice that week, being reassured by the fact that she would visit her lymphedema therapist that day who “would also examine her breasts during therapy.”

The therapist explained that exposure exercises could temporarily increase anxiety as NG worked on decreasing her excessive attempts to self-control her own emotions and behavior. NG recognized that these moments of exposure also offered opportunities to practice cognitive reframing and to generate more helpful beliefs.

#### Session 5: Face-to-Face

NG felt more in control of her fears and was no longer stuck in a negative thought spiral. Although hearing or reading about cancer still triggered cancer-related thoughts, she felt better equipped to reframe them into more helpful ones. For instance, NG had attended her sister’s wedding anniversary where a relative told her about his medical problems (coughing up blood) and the medical examinations he had had. Although NG’s first thoughts were: “He has cancer…. and maybe even die of it.” she successfully replaced this belief by a more helpful one: “There’s no point in assuming the worst, we’ll just have to wait for his medical results.” NG did not find the relaxation assignments helpful and the willingness to practice had further declined. The therapist decided it would be best not to pursue relaxation exercises any further.

Prior to therapy NG performed breast exams daily. Now, 2 months later, she felt confident enough to stop breast self-exams completely as long as she regularly visited the lymphedema therapist (a safety behavior). NG and the therapist decided to work towards completely ending lymphedema therapy and mutually agreed that, after the last lymphedema therapy session, NG could examine her breasts monthly (at most) as recommended by Dutch breast self-examination guidelines.

From therapy, NG learned that sharing her concerns brings relief. While she had always considered herself talkative, fear of rejection or disapproval prevented her from expressing her feelings and sharing her thoughts with others, so that she kept her fears to herself. At this point, NG said she would like to involve her husband in her process of change in order to strengthen their relationship.

#### Sessions 6 and 7: Conjoint Therapy with Partner Present (Instead of Telephone Contact)

In the original protocol for treatment, session 6 and session 7 each were planned to be a 15-min one-on-one telephone contact. However, that was changed by events in session 5, which led to a mutual decision to have NG’s partner join the session and the therapist seeing NG and her partner together. In session 5, the previous session, NG had expressed the feeling that it would be good for her to share her concerns with others. However, she found FCR difficult to discuss with family members as she did not want to burden them. Yet she also experienced feelings of guilt from not sharing her concerns with her partner (“It would be nice to hear what my partner thinks and good for him to hear what goes on in my head”). Therefore, the therapist suggested to NG that it could be beneficial to invite her partner to session 6 and 7 for a conjoint therapy meeting. This would create an opportunity to improve communication, mutual trust, and openness between the partners and to give NG’s partner more insight into the depths of the problem NG was experiencing. Furthermore, her partner would be able to provide his views on FCR and on how NG is doing in daily life. For these reasons, a deviation from treatment protocol was made and her partner attended session 6 and 7.

NG was increasingly more able to look to a future beyond cancer. She felt confident enough to plan future activities and formulate future goals in terms of weeks, months, and years. She planned two vacations abroad, something she had not dared to do in the past 2 years because of her FCR. By the end of session 7, NG was more proficient in replacing negative thoughts with more helpful ones: “I do not know what the future will bring in terms of health, but worrying is not going to help and I do not want the rest of my days to be overshadowed by doubt.”

At the end of session 7 NG and her partner, both agreed that it was not needed for her partner to join the final session. They felt that they had achieved their mutual goals and therefore initiated the ending of the conjoint treatment sessions. NG’s partner expressed that he had found it useful to join the two sessions as it provided him with more insight in the process of change NG was going through. NG felt supported by her partner: “It feels like therapy has brought us closer to each other. We have become more able to talk together.” For instance, NG’s partner had helped her to form more pragmatic future goals such as planning a holiday together.

#### Session 8: Face-to-Face

NG attended this session individually. The past 2 weeks she had been working on a relapse prevention plan and in session 8 the relapse prevention plan was reviewed and further refined. One of the first steps for NG was to learn to identify her “red flags”: situations that left her more vulnerable to a FCR relapse. Situations included periods of sickness and general stress. Secondly, the therapist helped NG to come up with a list of warning signs that may indicate a nearing relapse: more nervousness, more arguments with her husband, feelings of sadness, avoiding more social activities, and not wanting to plan activities ahead. Next, NG identified strategies that could help her manage her fears: “I should not beat myself up, I should stop that train of thoughts from running wild. Just stop everything for a second… Distance myself from the situation… Analyze what is going on… And maybe discuss my fears with my husband as well.” One of her top priorities was to continue practicing her CBT skills.

NG felt she had enough tools to work on her fears after completion of therapy and that control over FCR had increased. She now examined her breasts monthly and felt confident enough to end lymphedema therapy.

#### Session 9: Face-to-Face 3-Month Follow-Up

NG and her husband attended the 3-month follow-up session. NG reported that she was doing well and no longer struggled with FCR. She had come to accept that FCR is a normal emotion that presents itself in certain situations and was no longer overwhelmed by her fears. NG was satisfied with the process and outcomes of therapy and found that completing thought records was the most helpful part of therapy. She felt better equipped to deal with FCR in daily life and was able to use learned skills and techniques to cope with situations that trigger FCR. Lymphedema therapy had been completed and NG no longer felt the strong need to examine her breasts. Both therapist and patient decided that no further contact was needed. At the third assessment, 6 months after completion of therapy, 3 weeks before her annual mammography, NG reported not being overwhelmed by her fears, but instead, she felt in control.

## Results

### Quantitative Therapy Evaluation

#### Perceived Control Over FCR

**Fig. 2:**
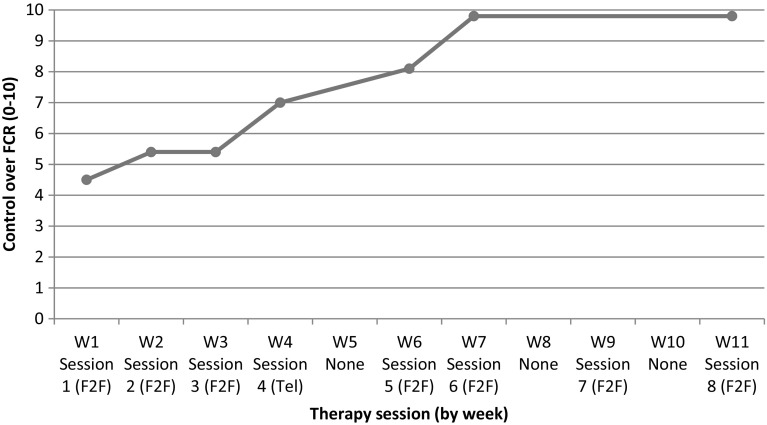
Perceived control over FCR during therapy. *W* week, *F2F* face-to-face meeting, *Tel* telephone meeting

During treatment, the patient’s sense of control over FCR increased to almost maximum (Fig. 2).

#### Severity and Other Dimensions of FCR

There was a marked decrease in rated FCR severity from T0 to T1, i.e., FCR severity went from a clinically elevated level to a lower, non-clinical level. However, after T1, FCR severity appears to have remained at a stable, non-clinical level of FCR. FCRI scores for severity, triggers, and insight showed reliable improvement at all time points (Table 2).

**Table 2:** Fear of cancer recurrence at four times and compared to mean scores of breast cancer survivors (BCS)

	Range	Screening	T0	T1	T2	T3	BCS M(SD)
CWS^a^	(8–32)	25	21	14^c^	10^c^	10^c^	13.4 (3.9)
FCRI^b^		–					
Severity	(0–36)	–	26	13^c^	10^c^	12^c^	14.3 (7.6)
Triggers	(0–32)	–	22	10^c^	12^c^	12^c^	13.6 (6.9)
Distress	(0–16)	–	10	12	2^c^	4^c^	5.4 (3.8)
Func impairment	(0–24)	–	8	3	0^c^	0^c^	3.1 (4.1)
Insight	(0–12)	–	7	4^c^	4^c^	0^c^	1.7 (2.4)
Reassurance	(0–12)	–	6	6	5	4	3.2 (2.9)

^a^Dutch breast cancer survivors

^b^Canadian breast cancer survivors

^c^RCI value exceeds > 1.96 and indicates reliable change

#### (Breast Cancer-Specific) QoL

Physical functioning, emotional functioning, and GH/QoL scores had increased post-treatment (T1) and remained high at 12-month post-treatment (T3, Table 3). The symptom scales Fatigue and Insomnia were lower at all follow-up assessments (T1 to T3) compared to baseline (T0).

**Table 3:** Quality of life scores at four times compared to median scores of breast cancer survivors

	T0	T1	T2	T3	BCS^b^ Mean (SD)
QLQ-C30^a^					
Physical Functioning	73	93	80	80	76.9 (21.6)
Role Functioning	100	100	100	100	71.0 (31.1)
Emotional Functioning	66	100	100	100	71.0 (23.4)
Cognitive Functioning	100	83	66	83	82.6 (21.1)
Social Functioning	100	100	100	100	81.5 (25)
GH/QoL	75	100	83	100	62.2 (24.4)
Fatigue	22	0	0	11	33.4 (26.1)
Nausea	0	0	0	0	7.6 (18.3)
Pain	0	0	0	0	28.7 (29.5)
Dyspnea	0	0	0	0	19.0 (28.5)
Insomnia	100	66	66	66	28.1 (32.3)
Appetite	0	0	0	0	17.6 (28.5)
Constipation	0	0	0	0	17.4 (27.4)
Diarrhea	0	0	0	0	6.2 (17.5)
Financial	0	0	0	0	14.3 (25.5)
QLQ-BR23^a^					
Body Image	50	58	75	66	85.0 (21.7)
Sexual Functioning	–	33	33	–	11.3 (17.8)
Sexual Enjoyment	–	66	66	–	49.1 (25.8)
Future Perspective	33	66	100	100	52.4 (34.3)
Breast Symptoms	33	8	16	25	16.2 (16.9)
Arm Symptoms	0	0	0	0	20.8 (21.9)

^a^Range is 0–100 for all scales

^b^Reference values of a multi-ethnic reference group of 523 female breast cancer survivors, aged between 60 and 69 years (Scott et al., 2008)

Future perspective increased between pre-treatment (T0), post-treatment (T1), and 6-month follow-up (T2) after which it remained stable. Body image and sexual functioning/enjoyment varied over time. Breast symptoms reduced after therapy but increased at follow-up (T2 and T3).

Compared to a BCa specific norm population, NG scored within the normal range (mean ± 1SD) on all QLQ-C30 functioning scales and on the GH/QoL scale. She did, however, score high on complaints of insomnia at all time-points. Regarding the QLQ-BR23 scales, NG reported a worse (mean ± 1SD) body image at baseline (T0) and directly post-treatment (T1) but her body image was comparative at 6- and 12-month follow-up (T2 and T3). Regarding her future perspective, NG scored higher than the normative group (mean ± 1SD) on both follow-up time points (T2 and T3).

#### Exploratory Outcomes

NG was clinically distressed (DT, IES) before therapy, scores fell within the normal range at all times after therapy (Table 3). Changes were evident on both the IES avoidance and intrusion subscales. Distress and fatigue had decreased at 12-month post-therapy (T3), while optimism and satisfaction with life had increased compared to pre-therapy (T0). NG completed 32/33 assignments, indicating good adherence (Table 4).

**Table 4:** Exploratory outcomes at T0 through T3

	Range	T0	T1	T2	T3
Distress thermometer					
Thermometer	(0–10)	4	0	0	0
VVV					
Fatigue	(4–32)	13	5	7	7
HADS					
Total	(0–42)	9	2	5	1
HADS-anxiety	(0–21)	8	0	4	1
HADS-depression	(0–21)	1	2	1	0
IES					
Total	(0–75)	38	2	7	0
Intrusion	(0–35)	24	1	5	0
Avoidance	(0–40)	14	1	2	0
SWLS					
Total	(5–35)	30	31	33	32
LOT					
Total	(0–32)	16	22	19	22
BVS					
Attentional focus	(0–45)	14.3	6.13	18.01	4.26

### Qualitative Nurse Evaluation

Five months after NG had completed therapy, she was seen by the nurse specialist for her annual medical check-up. Two weeks before this appointment, NG had completed a DT at home that she brought with her to the consultation. The DT did not show signs of distress, anxiety, or tension. While NG told the nurse that their appointment did trigger FCR, she now felt better equipped to manage her fears. NG did not request any extra medical examinations, nor did she contact the nurse outside their regular appointments. She now examined her breasts monthly.

## Discussion

This case study provides an intensive overview of the course and content of CBT for a highly fearful breast cancer survivor. Comparison of the pretest, post-test, and follow-up scores showed that FCR severity decreased to non-clinical levels after completion of CBT. Improvement was still evident at 6- and 12-month post-therapy. Several exploratory outcomes improved to the level of non-fearful BCa controls. Self-perceived control over FCR increased sharply after the third therapy session. While speculative, a possible reason for this is that NG reported benefit from the home assignments on cognitive therapy, which was a central aspect of therapy in the second and third session. Lymphedema therapy contributed to high FCR as it was both a trigger of FCR (being reminded of the disease and its treatment) as well as a safety behavior (“If anything feels off in my breast, the therapist will notice”). Working towards ending this therapy and normalizing breast self-exams may have also played a role in NG’s improvement.

Even though CBT is manualized, this study illustrates how important it is to tailor treatment to a patient’s needs. In the first therapy session, a personalized FCR model was established that served as a guide for selecting treatment components. By visualizing the information from the intake assessment in a personal model, the therapist identified intrusive cognitions and excessive controlling behavior as two important processes maintaining FCR. Furthermore, NG wanted to involve her partner in her therapy. The therapist agreed that this could have a beneficial effect on NG’s process of change. The next two sessions, which should have been telephone consultations, were instead face-to-face consultations so that both NG and her partner could be present. Inflexible adherence to the manual would not have addressed the marital concerns that also played a role in maintaining NG’s FCR. Finally, NG experienced emotional discomfort practicing relaxation exercises at home, even in comfortable circumstances. After practicing for 3 weeks, NG indicated that she still experienced discomfort and thereby expressed a strong preference not to practice relaxation anymore. The therapist therefore decided it would be best for the remaining sessions to focus on treatment components more beneficial for therapy progress, such as cognitive restructuring.

NG was the first high fearful cancer survivor referred to medical psychology unit since the CBT program became available; she was therefore selected for this case study. Based on this pilot case, two changes have been made to the finalized treatment protocol: (1) partners are now allowed (and invited) to join the patient for all face-to-face sessions from session five onwards, and (2) assignments on identifying personal strengths and resources of strength to deal with FCR have been moved from session 6 to session 5. The main reason for both adjustments is that social support (and especially partner support) seems to play an important role in influencing FCR and should therefore be addressed in a timely manner (Crist & Grunfeld, 2013; Koch et al., 2013). NG had an excellent response to CBT and there were no complicating factors: she was highly motivated, showed no treatment resistance, was not taking any psychotropic medications and had near excellent homework adherence. We assume that other patients, especially those with a more avoidant way of coping, might show more initial resistance and may find it harder to complete the home-assignments.

Since this was an uncontrolled case study, it is not known whether the amelioration of clinical FCR was due to treatment or spontaneous remission over time. Nevertheless, a review by Simard et al., reported that 18 (out of 22) longitudinal studies did not find a spontaneous change of FCR over time in the post-treatment period (range 3 months–6 years of post-treatment; Simard et al., 2013). Thus, when no intervention is offered, FCR seems to be a relatively stable and persistent problem over time (Savard & Ivers, 2013; Simard et al., 2013). Our case was diagnosed with BCa more than 2 years before our intervention; since cancer treatment ended, nurse reports made mention of persistent FCR. Thus, while we cannot rule out “time” as a factor, the literature favors the possibility that the decline in FCR we found may be attributable to our intervention. Given the complexity of most psychotherapeutic interventions, it is hardly possible to delineate the most effective treatment components of CBT for clinical FCR. Treatment gains may also be attributable to non-specific factors, such as therapist attention and good therapeutic rapport.

To conclude, this study demonstrates one way of using CBT for managing high FCR in cancer survivors. The feasibility and efficacy of current intervention have been investigated in a large-scale randomized controlled trial (the SWORD-study) where it was found that the treatment program had both a statistically and clinically significant effect on the severity of FCR in breast, prostate, and colorectal cancer survivors (van de Wal et al., 2017).

## Authors Respond to Questions Concerning the Case of NG: Judith Prins, Clinical Psychologist, Treatment Supervisor, and Researcher; Petra Servaes, Clinical Psychologist and Treatment Provider; Belinda Thewes, Clinical Psychologist and Researcher and Marieke van de Wal, Psychologist and Researcher

### Issue 1. In This Case, CBT was Used to Reduce FCR Severity in a Cancer Survivor. FCR can also be a Concern for Patients with a Different Chronic Disease (e.g., Fear of Progression in Diabetes). What are the Authors’ Thoughts About Using This Therapy for Treating Fears Associated with Other Somatic Conditions?

Response. *Judith Prins, Petra Servaes, Belinda Thewes, Marieke van de Wal*: We know from both clinical experience and scientific work that patients suffering from various chronic conditions, such as multiple sclerosis, heart disease, rheumatoid arthritis, and diabetes mellitus, also experience fear of disease progression (Dankert et al. 2003). However, the manifestation of fear in patients with chronic diseases is varied and depends largely on the natural course of the disease (e.g., progressive deterioration vs. intermittent relapses with deterioration over time vs. potentially curable relapses). For instance, for those diagnosed with type 1 diabetes mellitus know that their health will gradually decrease over a 20- to 30-year period and that serious health problems, such as kidney or eye complications and neuropathy, are almost inevitable. Whereas for cancer patients, the pattern of disease progression is more varied, with some experiencing relapse followed by a rapid deterioration, or intermittent relapse, or potentially curable relapse. Whilst the general elements of this therapy, such as self-monitoring, cognitive restructuring, behavioral modification, and mindfulness, could be generalized to treat fear of disease progression in other chronic somatic conditions, the specific content of the assignments and psycho-educational material should be tailored to make it disease specific.

Follow-up question. Relating to the issue above, would this intervention also be applicable to cancer patients who are not disease-free but already have a recurrence and experience fear of progression?

Response. Yes. Most of the techniques could be applied to fear of progression in currently ill cancer patients as well (Thewes et al. 2017). Group CBT has been demonstrated to help cancer patients with a progressive disease cope with fear of cancer progression (Herschbach et al., 2010). Whilst it is likely that techniques contained within the described intervention would be helpful for patients with progressive disease, the content of the intervention would require adaptation. For example, in a palliative setting there may also be the need to add additional themes, such as working towards acceptance of one’s inevitable death, dealing with existential concerns and planning for end-of-life care. These are topics not covered in our manualized CBT for FCR in disease-free cancer survivors.

### Issue 2. You Highlight an Interesting “Gray Area” in Which it Might be Difficult to Differentiate Adaptive Health Behaviors from Safety Behaviors (In This Case, Visiting a Lymphedema Therapist). Could You Further Elaborate on This?

Response. *Judith Prins, Petra Servaes, Belinda Thewes, Marieke van de Wal*: There is a fine line between health behavior that is considered adaptive and behavior that can be classified as maladaptive. While there is nothing wrong with a patient being vigilant, the problem with FCR is that highly fearful survivors tend to become hypervigilant. CBT for FCR promotes body awareness and vigilance by teaching patients appropriate self-management skills (e.g., carrying out breast self-exams according to the frequency advised by the oncology guidelines), and by providing them with mindfulness and relaxation exercises. However, we discourage excessive reassurance seeking behavior whereby patients repeatedly visit health care providers to ask about their symptoms.

Most cancer patients will require medical attention during follow-up care and some will be instructed to perform regular self-checks (for example breast self-examination). The goal of treatment is to encourage patients to get the balance right.

In the case of NG, initially the focus of her lymphedema therapy was to receive manual lymph drainage to reduce swelling of the breast. However, once her lymphedema was managed, visiting the lymph edema therapist became a safety behavior and a means for NG to seek medical reassurance that her cancer had not recurred. At this point, seeking additional appointments with the lymphedema therapist became maladaptive; it provided NG with only temporary relief from FCR and did not alleviate her concerns in the long run. When differentiating unhelpful from helpful medical reassurance seeking, therapists should not only explore whether the frequency is in excess of what is recommended, but also the patients’ perception of why they are consulting a health professional, and whether seeing the patient would be going beyond the provider’s appropriate professional role. For example, patients should not visit a lymphedema therapist to be reassured about their concerns for a recurrence.

Follow-up question. In this case study, FCR was not only related to visits to the lymphedema therapist but initially also with increased contact moments with the nurse specialist. Could you comment further on FCR from the perspective of healthcare service utilization? How does this factor into the value of CBT and other therapies for FCR?

Response. *Judith Prins, Belinda Thewes, Marieke van de Wal*: We know that there are mixed findings on the relationship between FCR and healthcare service utilization. There is evidence that cancer survivors with high FCR are more likely to refuse discharge from a cancer center (Glynne-Jones et al. 1997), are less satisfied with their care (Hart et al. 2008), and are more likely to seek readmission to a cancer center (Glynne-Jones et al. 1997), which suggests that untreated high FCR is possibly associated with increased healthcare costs. However, some highly fearful patients adopt an avoidant way of coping and may avoid healthcare services medical check-ups that may trigger FCR (Sarkar et al. 2015). Documenting evidence of avoidance is difficult as avoidant patients may also avoid participating in research about FCR. A recent review on the cost-effectiveness of psychosocial interventions for cancer survivors concluded that CBT for psychosocial problems in cancer survivors yields good value for money (Dieng et al. 2016). Also, one study demonstrated the cost-effectiveness of CBT over a supportive-experiential group in targeting fear of progression (Sabariego et al. 2011). We believe that it would therefore be interesting to look further into the cost-effectiveness of CBT for FCR.

### Issue 3. Regarding the Authors’ Decision to Invite Partners to One or Multiple Therapy Sessions: Why was This Done? Please Elaborate

Response. *Judith Prins, Petra Servaes, Belinda Thewes, Marieke van de Wal*: Cancer has an impact on both patient and partner (van de Wal et al. 2017). In the treatment of cancer-related psychological problems, like FCR, it can therefore be very useful to involve both parties.

Partners are often very dependent on the patient for information about the patient’s current health status and the related subjective risk of recurrence. They have little knowledge of what is going on in a patient’s mind or body. Therefore, partners cannot apply the same coping strategies to deal with FCR as the patient does, such as self-monitoring of symptoms. Partners often question the patient: “How do you feel? Are you sure everything is okay?” If this happens regularly, it may further heighten the patient’s FCR. Many cancer patients report not disclosing fears either because they do not like to share their feelings or because they do not want to burden their loved ones. However, a lack of discussion about FCR between couples may actually intensify FCR for both the patient and the partner. Thus, we believe it is important to always identify what the role of the partner is in maintaining FCR, and if needed, consider inviting the partner to join one or two sessions.

### Issue 4. NG was Referred by R. Berry, the Nurse Specialist. Do You have Any Recommendations for Healthcare Providers About When to Refer Patients to Receive Additional Care for FCR?

Response. *Judith Prins, Belinda Thewes, Marieke van de Wal*: We know from an earlier study that healthcare professionals find it very difficult to identify FCR and that they also struggle with knowing how to deal with it (Thewes et al. 2013). It is normal for patients to have concerns about a possible recurrence during the survivorship trajectory and especially when confronted with medical examinations and follow-up consultations related to cancer. To differentiate normal FCR from high FCR, it is therefore important for healthcare providers to use objective measures to screen for high FCR. Although there is no consensus on which screening instrument is best, several brief measures are available as described in an extensive literature review of quantitative measures of FCR (Thewes, Butow, Zachariae, et al., 2012). If no validated screening instrument is available, basic questions may also be sufficient. Additionally, healthcare providers should be aware of reassurance seeking or avoidant behavior that might indicate high FCR, such as frequent unscheduled telephone contacts or canceling appointments. Finally, professionals should inform themselves of local resources available to help patients that struggle with high FCR and how to refer patients to them.

### Issue 5. In This Therapy You have Used a Manualized Intervention, Which was Very Fixed in Structure. Yet You have Decided to Change the Protocol by Inviting NG’s Partner and Leaving Out the Mindfulness Exercise. What is Your Opinion on Strictly Following Protocols Versus A More Tailored Approach in Which You are More Flexible

Response. *Judith Prins, Petra Servaes, Belinda Thewes, Marieke van de Wal*: In clinical studies, therapists should try to follow the protocol as closely as possible. The intervention was developed in a certain way and has been tested for efficacy; even minor protocol deviations may influence the therapy’s effectiveness. However, some patients might not fit the protocol and require deviations. Deviations inevitably occur and then it is important to document them and to discuss them within your team.

We believe that in clinical practice, therapeutic protocols should take advantage of evidence-based treatment procedures or manuals that are available. In clinical practice, therapeutic protocols should be treated as guidelines that should be personalized if needed. Rigid adherence to a standardized and empirically validated treatment protocol may enhance treatment fidelity but it does not necessarily offer the best therapy. It may deny and discount emotional experiences that are important for the patient and may negatively affect the therapeutic alliance. For instance, NG did not wish to continue mindfulness practice and the therapist respected that decision.

### Issue 6. Do You Think it is Realistic and Feasible to Implement This Intervention in Clinical Practice if Future Research Demonstrates the Same Positive Results as Found in This Study? Are There Any Specific Barriers or Pitfalls?

Response. *Judith Prins, Petra Servaes, Belinda Thewes, Marieke van de Wal*: If proven efficacious, we plan to conduct a formal implementation study of this intervention including an analysis of barriers and facilitators for implementation. We know from earlier studies and our own experience that the implementation of psychosocial interventions outside of academic centers is possible and necessary, but there will be barriers that have to be dealt with. For instance, the personnel involved may lack the required time to receive adequate protocol training or supervision, there is diffusion of responsibility and financial constraints can be an issue. The effect size is often lower when an intervention is tested outside a specialized centre where a small number of therapists are highly trained and closely supervised (Schreurs et al. 2011). Furthermore, outside of research settings, patients are less carefully screened and selected. Patients in randomized controlled trials are often a selected group that does not necessarily reflect the diversity of patient characteristics seen in the broader population. When this intervention is implemented more broadly, and the treatment is applied to a more diverse population of patients, some further tailoring of the intervention is likely to be required. CBT has already been used to treat FCR in a number of breast, prostate, and colorectal cancer survivors (van de Wal et al. 2015, 2017). Therapist evaluations in using CBT to treat FCR were very positive. One of the things they have mentioned is that patients appreciate the cancer-specific content of the intervention. Our treatment includes videos and examples on all three cancer types so patients can easily identify with the patient models. Prior to implementation, it may be necessary to include a greater diversity of cancer types in the videos and exercises.

Patients’ perception of their situation may also create barriers to successful implementation. FCR is a sensitive topic, and patients, themselves, may be reluctant to address the topic because they consider it to be taboo. They may feel that they should not burden their healthcare provider, and so try to self-manage their concerns about a recurrence. An important task for practitioners is to discuss FCR during clinical visits, and in case of suspected high FCR, consider referring patients for counseling.
